# Neuroprotective effects of salidroside against cerebral ischemia-reperfusion injury involve downregulation of total NAMPT and suppression of microglial inflammation

**DOI:** 10.3389/fphar.2026.1800667

**Published:** 2026-03-31

**Authors:** Meng Zhang, Junchao Xue, Yilin Ping, Huimin Li

**Affiliations:** 1 Department of Clinical Laboratory, Tongde Hospital of Zhejiang Province, Hangzhou, Zhejiang, China; 2 Department of Pharmacy, Tongde Hospital of Zhejiang Province, Hangzhou, Zhejiang, China; 3 Department of Stomatology, Liangzhu Community Health Service Center, Hangzhou, Zhejiang, China

**Keywords:** cerebral ischemia-reperfusion, microglia, NAD+, neuroinflammation, nicotinamide phosphoribosyltransferase expression, salidroside

## Abstract

**Objective:**

This study investigated the protective effect of Salidroside (SAL) against cerebral ischemia-reperfusion (I/R) injury and its role in regulating nicotinamide phosphoribosyltransferase (NAMPT)-mediated neuroinflammation and damage.

**Methods:**

*In vivo*, a middle cerebral artery occlusion/reperfusion (MCAO/R) model was established in male SD rats. Animals were divided into sham-operated (Sham), I/R (MCAO-NS), MCAO+eNAMPT, and MCAO+eNAMPT+SAL groups. Recombinant NAMPT (5 μg/rat) and/or SAL (10 μg/rat) were administered intracerebroventricularly. Neurological deficit scores (mNSS), infarct volume (TTC), brain levels of IL-1β/TNF-α (ELISA), total NAMPT expression (WB/ELISA), and NAD+ content were assessed. *In vitro*, primary microglia were subjected to oxygen-glucose deprivation/reoxygenation (OGD/R) and divided into control (Ctrl), OGD-NS, OGD+eNAMPT (20 ng/mL), and OGD+eNAMPT+SAL (6 μg/mL) groups. Cytotoxicity (LDH), viability (MTT), and IL-1β/TNF-α secretion (ELISA) were measured.

**Results:**

Compared to Sham, the I/R group showed worsened neurological deficits, larger infarct volumes, elevated total NAMPT, TNF-α, and IL-1β levels (*P* < 0.001), and reduced NAD+ (*P* < 0.001). Exogenous eNAMPT further exacerbated these injuries and inflammation (*P* < 0.001). SAL treatment significantly reversed eNAMPT-aggravated neurological deficits and infarction (*P* < 0.001), downregulated total NAMPT, TNF-α, and IL-1β, and increased NAD+ levels (*P* < 0.001). *In vitro*, eNAMPT stimulation increased OGD/R-induced TNF-α and IL-1β secretion from microglia (*P* < 0.001), which SAL effectively inhibited (*P* < 0.001).

**Conclusion:**

This study provides experimental evidence that the neuroprotective effects of SAL against cerebral I/R injury are associated with downregulation of pathologically elevated NAMPT expression (likely reflecting a reduction in pro-inflammatory eNAMPT), restoration of cerebral NAD+ homeostasis (potentially preserving iNAMPT function), and suppression of microglia-mediated neuroinflammation, suggesting that eNAMPT may serve as a potential effector molecule of SAL.

## 1 Introduction

Stroke is the second leading cause of death and the primary cause of adult disability worldwide, imposing a heavy burden on public health and the economy (Hilkens et al., 2024). With the accelerated aging of the population, its incidence continues to rise. It is projected that the number of stroke patients in China will exceed 30 million by 2030 (Hilkens et al., 2024; Saini et al., 2021). Ischemic stroke (IS) accounts for approximately 85% of all stroke cases. Its pathological core involves the interruption of cerebral blood flow leading to neuronal energy metabolism failure and subsequent cascade injury (Sarvari et al., 2020). Current standard clinical treatments primarily rely on intravenous thrombolysis (e.g., t-PA) and mechanical thrombectomy to achieve early recanalization (Sacks et al., 2018). However, these therapies have significant limitations: the therapeutic time window for t-PA is narrow (typically ≤4.5 h), and it is associated with risks of intracranial hemorrhage and neurotoxicity, preventing many patients from benefiting (Sacks et al., 2018; Emberson et al., 2014). More critically, blood flow recanalization often induces cerebral ischemia-reperfusion injury (CIRI), which further exacerbates brain tissue damage and has become a key limiting factor affecting patient prognosis (Zhou et al., 2023; Gomez et al., 2023).

The mechanisms of CIRI are complex, involving a vicious cycle of multiple processes including excitotoxicity, calcium overload, oxidative stress, mitochondrial dysfunction, and intense inflammatory responses (Gomez et al., 2023; Lin et al., 2023). Among these, neuroinflammation is considered a central driving force throughout the occurrence and progression of IS (Anrather and Iadecola, 2016). Following an ischemic event, microglia, as the resident immune cells of the central nervous system, are rapidly activated. They release a large number of pro-inflammatory factors such as tumor necrosis factor-alpha (TNF-α) and interleukin-1 beta (IL-1β), which directly damage neurons, disrupt the blood-brain barrier, and recruit peripheral immune cell infiltration, forming a continuously amplified inflammatory storm that ultimately exacerbates neurological deficits (Shen et al., 2023; Ajoolabady et al., 2021; Dai et al., 2020). Therefore, targeting and modulating excessive microglia-mediated neuroinflammation has become a crucial therapeutic strategy for alleviating CIRI.

NAMPT is the rate-limiting enzyme in the salvage pathway for nicotinamide adenine dinucleotide (NAD^+^) biosynthesis in mammalian cells, playing a central role in maintaining cellular energy metabolism and redox homeostasis. Recent studies indicate that NAMPT exhibits a “dual function” under pathological conditions: on one hand, its intracellular form (iNAMPT) is crucial for maintaining neuronal NAD^+^ levels and supporting cell survival; on the other hand, it can be released as an extracellular form (eNAMPT), acting as a damage-associated molecular pattern (DAMP), being released in inflammatory diseases such as ischemic stroke, and driving inflammatory cascades (Audrito et al., 2020; Martínez-Morcillo et al., 2021). Research shows that eNAMPT can promote the expression of pro-inflammatory factors such as TNF-α and IL-1β by activating pathways including nuclear factor-kappa B (NF-κB), thereby amplifying inflammation (Martínez-Morcillo et al., 2021; Fan et al., 2011).

During CIRI, brain total NAMPT expression is significantly upregulated, yet its role is controversial: iNAMPT in neurons exerts neuroprotective effects through its enzymatic activity, whereas eNAMPT secreted by activated glial cells exacerbates neuroinflammation and brain injury in a non-enzymatic manner (Wang et al., 2019; Zhao et al., 2015; Zhao et al., 2013). As microglia are central drivers of neuroinflammation, whether their activation is directly regulated by eNAMPT remains incompletely understood. Therefore, elucidating the precise role and downstream mechanisms of NAMPT, particularly eNAMPT, in the fine-tuned regulation of microglial inflammatory responses is of significant importance for a deeper understanding of CIRI pathology and the development of novel therapeutic strategies.

SAL is the primary active component of the traditional Chinese medicine Rhodiola rosea, possessing multiple pharmacological activities including antioxidant, anti-inflammatory, and anti-apoptotic effects. It has demonstrated protective effects in cerebral ischemia models by alleviating neurological deficits and reducing infarct volume (Fan et al., 2020; Zhang et al., 2021). The underlying mechanisms involve regulating the BDNF/PI3K/Akt survival pathway, activating the Nrf2 antioxidant pathway, and inhibiting NF-κB/NLRP3-mediated neuroinflammation, among others (Zhang et al., 2018; Han et al., 2015). Notably, SAL can significantly modulate microglial phenotypes by suppressing their pro-inflammatory M1 polarization, thereby mitigating post-ischemic neuroinflammation (Liu et al., 2018). This characteristic is highly relevant to the pivotal role of eNAMPT in CIRI. Our preliminary studies have confirmed that exogenous eNAMPT can exacerbate cerebral ischemic injury through a non-enzymatic mechanism and may be involved in the inflammatory activation of microglia (Zhao et al., 2013). However, it remains unclear whether the neuroprotective effects of SAL are associated with modulation of the eNAMPT-driven inflammatory pathway or its impact on iNAMPT-mediated NAD^+^ metabolism.

Therefore, this study aims to investigate whether the neuroprotective effects of SAL are associated with changes in total NAMPT expression and its functional consequences, distinguishing between its potential effects on eNAMPT-driven inflammation and iNAMPT-mediated NAD^+^ metabolism. By systematically examining the impact of SAL on NAMPT expression, NAD^+^ metabolic homeostasis, and microglia-mediated neuroinflammation, this research seeks to provide new experimental evidence for understanding the pharmacological actions of SAL. It is anticipated that these findings will offer new insights for the clinical application of SAL and the development of therapeutic strategies for ischemic stroke.

## 2 Materials and methods

### 2.1 Animals and ethical statement

Adult male Sprague-Dawley rats (8 weeks old, 300–320 g) were obtained from the Experimental Animal Center of Hangzhou Medical College (Permit No. SYXK 2014-0008). Animals were housed under standard conditions (22 °C ± 1 °C, 55%–65% humidity, 12-h light/dark cycle) with free access to food and water. All experimental procedures were approved by the Animal Ethics Committee of Hangzhou Medical College and conducted in accordance with the National Institutes of Health Guide for the Care and Use of Laboratory Animals.

### 2.2 Experimental groups

*In Vivo*:

① Sham-operated group: Serving as the control group, only the carotid artery was exposed without occlusion.
② Ischemia-reperfusion group: A rat model of focal cerebral ischemia was established by middle cerebral artery occlusion (MCAO) followed by reperfusion surgery.
③ Ischemia-reperfusion + eNAMPT group: A rat model of focal cerebral ischemia was established by MCAO/reperfusion surgery. The rats received an intracerebroventricular injection of recombinant NAMPT protein (referred to as exogenous eNAMPT; concentration: 1 μg/μL; single dose: 5 μg/rat; injection volume: 5 μL).
④ Ischemia-reperfusion + eNAMPT + SAL group: A rat model of focal cerebral ischemia was established by MCAO/reperfusion surgery. The rats received an intracerebroventricular injection of both recombinant NAMPT protein (exogenous eNAMPT; concentration: 1 μg/μL; single dose: 5 μg/rat; injection volume: 5 μL) and SAL (SAL concentration: 2 μg/μL; single dose: 10 μg/rat; injection volume: 5 μL).

To precisely validate the central pharmacological action of SAL and its direct modulation of the NAMPT-mediated neuroinflammatory axis within the brain parenchyma, thereby circumventing the potential confounding factors of the blood-brain barrier (BBB) and peripheral drug metabolism or immune responses, all *in vivo* administrations in this mechanistic study were performed via the intracerebroventricular (ICV) route.

*In vitro* (Primary microglia):

① Ctrl group: Cells were cultured under normal conditions without any treatment.
② OGD-NS group: A cellular oxygen-glucose deprivation and reoxygenation (OGD/R) model was established.
③ OGD + eNAMPT group: The OGD/R model was established, and the cells were stimulated with recombinant NAMPT protein (referred to as exogenous eNAMPT; concentration: 20 ng/mL).
④ OGD + eNAMPT +SAL group: The OGD/R model was established, and the cells were stimulated with both eNAMPT and SAL (SAL concentration: 6 μg/mL).

### 2.3 Methods

#### 2.3.1 Establishment of animal model

Rats were fasted for 8–10 h and then anesthetized by intraperitoneal injection of ketamine (100 mg/kg). Subsequently, an incision was made along the midline of the neck to fully expose and isolate the right common carotid artery (CCA), external carotid artery (ECA), and internal carotid artery (ICA). After isolation, the ECA was ligated and cut obliquely. A nylon monofilament suture (diameter 0.3 mm) was inserted into the ICA and advanced approximately 18–19 mm distally to occlude the origin of the middle cerebral artery (MCA). In sham-operated rats, the suture was inserted but not advanced to block the MCA. After 2 h of occlusion, the suture was slowly withdrawn to achieve reperfusion. Postoperatively, the rats were placed in a constant-temperature chamber maintained at 22 °C–25 °C, and body temperature was regulated using a heating lamp. Neurological function was evaluated using the modified Neurological Severity Score (mNSS) by two independent observers who were blinded to the experimental groups.

#### 2.3.2 Intracerebroventricular injection

Following deep anesthesia, the scalp of the experimental rats was shaved and disinfected with povidone-iodine and 75% ethanol. The animals were then securely positioned on a stereotaxic frame. A microinjection syringe was vertically lowered into the right lateral ventricle at coordinates 1.0 mm posterior to the bregma, 2.0 mm lateral to the midline, and to a depth of 3.5 mm. The prepared solutions—exogenous eNAMPT, SAL—were administered via slow microinjection into the lateral ventricle.

#### 2.3.3 Measurement of cerebral infarct volume

TTC Staining for Infarct Volume Quantification: Following behavioral assessments, the rats were euthanized, and the brains were rapidly harvested. The brains were sectioned coronally into 2 mm thick slices. These slices were incubated in a 0.5% solution of 2,3,5-triphenyltetrazolium chloride (TTC) (Sigma-Aldrich; Merck Group) at 37 °C for 30 min. Subsequently, the stained slices were fixed with 4% formaldehyde at room temperature for 24 h. The areas of the infarcted region and the contralateral hemisphere were measured using ImageJ software. To correct for the influence of brain edema on infarct volume, the following formula was used to calculate the corrected infarct volume percentage: Corrected infarct volume percentage = [(Area of contralateral hemisphere-Area of ipsilateral non-infarct region)/Area of contralateral hemisphere]×100%. Infarct volume is typically expressed as a percentage relative to the volume of the contralateral hemisphere.

#### 2.3.4 Establishment of the oxygen-glucose deprivation (OGD) cell model

Primary microglial cells in the logarithmic growth phase and in good condition were used. The culture medium was removed and replaced with pre-warmed glucose-free medium. The cells were then immediately placed into a sealed hypoxia chamber, which was flushed with a gas mixture of 95% N_2_ and 5% CO_2_. The chamber was then incubated at 37 °C for 4 h to simulate the ischemic phase.

#### 2.3.5 Protein expression detection

Western Blot Analysis: Proteins were extracted from brain tissue using radioimmunoprecipitation assay (RIPA) lysis buffer. A total of 30 µg of protein per sample was separated by sodium dodecyl sulfate-polyacrylamide gel electrophoresis (SDS-PAGE). The separated proteins were then transferred onto nitrocellulose membranes. The membranes were blocked with 10% skim milk at room temperature for 1 h. Following this, the membranes were incubated with primary antibodies at 4 °C overnight, including an anti-NAMPT antibody (which detects total NAMPT protein) and an anti-glyceraldehyde-3-phosphate dehydrogenase (GAPDH; 1:5,000) antibody. After washing with phosphate-buffered saline (PBS), the membranes were incubated with horseradish peroxidase (HRP)-conjugated secondary IgG antibodies (1:5,000) at room temperature for 1 h. Finally, protein bands were visualized using an enhanced chemiluminescence (ECL) Western blotting detection system, and band intensities were quantified and normalized to GAPDH expression levels.

#### 2.3.6 NAD+ level detection

The levels of NAD+ in brain tissue homogenates were measured using a commercially available NAD+/NADH assay kit according to the manufacturer’s instructions. Briefly, appropriate amounts of brain tissue homogenates were processed with extraction solution and centrifuged. The resulting supernatants were then incubated with the detection reaction mixture. Absorbance was measured at 450 nm using a microplate reader. The concentrations of NAD+ and NADH were calculated based on the standard curve, and the total NAD level as well as the NAD+/NADH ratio were subsequently determined.

#### 2.3.7 Cytotoxicity assay

The cytotoxicity of primary microglia was evaluated by measuring lactate dehydrogenase (LDH) release into the culture supernatant using a commercial LDH Cytotoxicity Assay Kit, following the manufacturer’s instructions. Briefly, the culture supernatant from each experimental group was collected, centrifuged, and a volume of the resulting supernatant was mixed with the LDH assay working solution in a 96-well plate. The reaction was incubated in the dark. Absorbance was then measured at 490 nm using a microplate reader. The actual LDH release in each sample was calculated based on a standard curve, providing an assessment of the degree of cell damage and cytotoxicity.

#### 2.3.8 ELISA for detecting NAMPT, TNF-α, and IL-1β levels

Brain tissue homogenates or cell culture supernatants were collected. The concentrations of total NAMPT, TNF-α, and IL-1β were determined using commercially available enzyme-linked immunosorbent assay (ELISA) kits (Enogene, Mississauga, Ontario, Canada) according to the manufacturer’s instructions. All absorbance values were converted to corresponding concentration values based on the standard curve.

### 2.4 Statistical analysis

Data are presented as mean ± standard deviation (SD). Normality of data distribution was assessed using the Shapiro-Wilk test, and all data were confirmed to be normally distributed (*P* > 0.05). Statistical analysis was performed using GraphPad Prism 8.0 software. Comparisons between two groups were conducted using Student’s t-test. For comparisons among multiple groups, one-way analysis of variance (ANOVA) was employed, followed by Tukey’s test for *post hoc* pairwise comparisons. Exact p-values are reported, with values less than 0.001 denoted as *P* < 0.001. A P-value < 0.05 was considered statistically significant.

## 3 Results

### 3.1 Cerebral ischemia-reperfusion upregulates intracerebral total NAMPT expression and triggers neuroinflammation

**FIGURE 1:**
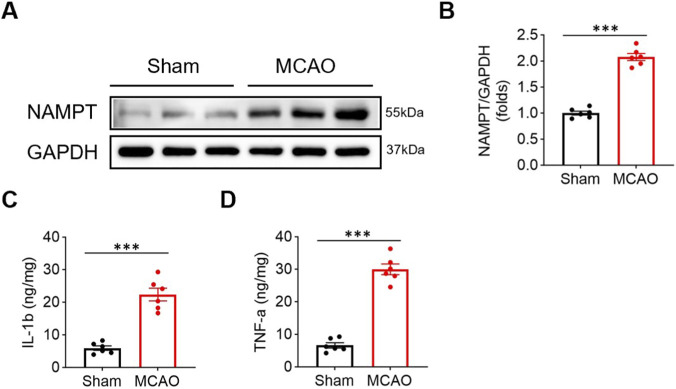
Upregulation of NAMPT and pro-inflammatory cytokines in rat brain after cerebral ischemia-reperfusion. **(A)** Representative Western blot bands showing total NAMPT and GAPDH protein expression in brain tissue from the sham-operated and MCAO/R groups. Due to the limited number of gel wells, three representative samples out of six per group are shown. Molecular weight markers were run concurrently with the samples but are not visible in the cropped image; the positions of target proteins (NAMPT 55 kDa, GAPDH 37 kDa) are indicated based on marker migration. **(B)** Densitometric analysis of NAMPT/GAPDH. Data are presented as mean ± SD (n = 6 per group). ***P < 0.001 vs. Sham group. **(C,D)** ELISA quantification of IL-1β **(C)** and TNF-α **(D)** levels in brain tissue. Data are presented as mean ± SD (n = 6 per group). ***P < 0.001 vs. Sham group.

To investigate the dynamic changes of NAMPT in cerebral ischemia-reperfusion (I/R) injury, we first examined the relevant molecular markers. Western blot analysis revealed that the expression level of total NAMPT protein in the ischemic cerebral cortex tissue of rats in the MCAO/R model group was significantly elevated compared to the sham-operated group (Relative expression: Sham group, 1.00 ± 0.09; MCAO group, 2.08 ± 0.17; t = 13.95, *P* < 0.001; Figures 1A,B). Consistently, ELISA results of pro-inflammatory cytokines in brain tissue homogenates showed that the levels of IL-1β and TNF-α in the model group were also significantly increased (IL-1β: Sham group, 5.94 ± 1.65 ng/mg; MCAO group, 22.36 ± 4.77 ng/mg; t = 7.97, *P* < 0.001; TNF-α: Sham group, 6.62 ± 1.99 ng/mg; MCAO group, 29.97 ± 3.99 ng/mg; t = 12.82, *P* < 0.001; Figures 1C,D).

These results suggest that cerebral I/R can significantly induce the upregulation of total NAMPT protein expression in the brain, accompanied by a robust neuroinflammatory response.

### 3.2 SAL alleviates exogenous eNAMPT-exacerbated cerebral ischemia-reperfusion injury

#### 3.2.1 SAL improves exogenous eNAMPT-induced increases in cerebral infarct volume and neurological deficit scores

**FIGURE 2:**
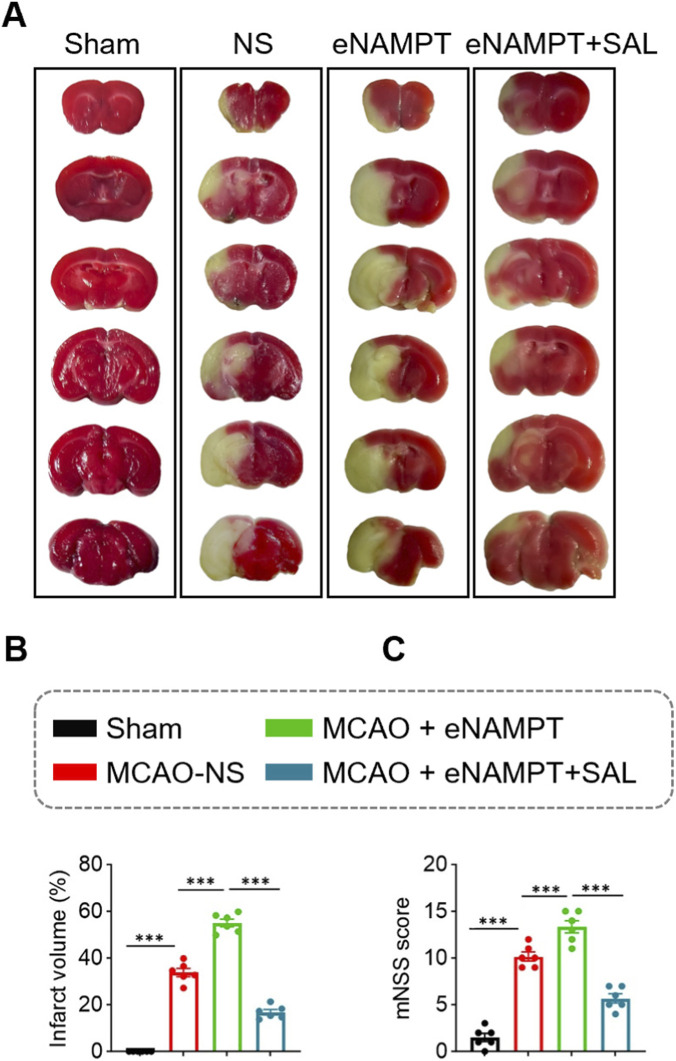
Comparison of cerebral infarct volume and neurological deficit scores among the four rat groups. **(A)** Representative brain sections stained with TTC from the four experimental groups. **(B)** Statistical analysis of cerebral infarct volume in the four groups. **(C)** Statistical analysis of mNSS scores in the four groups. Data are presented as mean ± SD (n = 6 per group). ***P < 0.001.

To investigate the effect of SAL on eNAMPT-mediated brain injury, we first assessed cerebral infarct volume and neurological deficit scores. Compared to the Sham group, both cerebral infarct volume and mNSS were significantly increased in the MCAO/R model group (MCAO-NS) (Infarct volume: 33.82% ± 4.28% vs. 0%, *P* < 0.001; mNSS: 10.17 ± 1.17 vs. 1.50 ± 1.05, *P* < 0.001). Intracerebroventricular injection of exogenous eNAMPT (MCAO + eNAMPT group) further exacerbated these injuries. Both infarct volume and mNSS in this group were significantly higher than those in the MCAO-NS group (Infarct volume: 54.98% ± 4.02% vs. 33.82% ± 4.28%, *P* < 0.001; mNSS: 13.33 ± 1.63 vs. 10.17 ± 1.17, *P* < 0.001). Treatment with SAL (MCAO + eNAMPT+ SAL group) significantly reversed this effect, reducing infarct volume by approximately 70% (to 16.72% ± 2.79%; *P* < 0.001 vs. MCAO + eNAMPT) and improving the neurological deficit score (5.67 ± 1.21; *P* < 0.001 vs. MCAO + eNAMPT) (Figures 2A–C).

#### 3.2.2 SAL inhibits exogenous eNAMPT-induced inflammatory response

**FIGURE 3:**
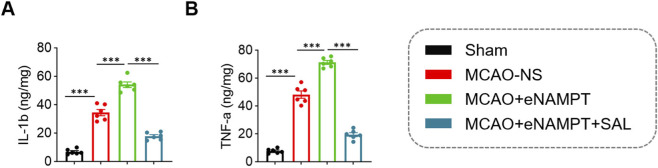
Comparison of inflammatory cytokine levels in brain tissue among the four rat groups. **(A)** Statistical analysis of IL-1β content in brain tissue across the four groups. **(B)** Statistical analysis of TNF-α content in brain tissue across the four groups. Data are presented as mean ± SD (n = 6 per group). ***P < 0.001.

Exogenous eNAMPT exacerbated the post-ischemic inflammatory response. Compared to the MCAO-NS group, the levels of pro-inflammatory cytokines IL-1β and TNF-α in brain tissue homogenates were significantly further elevated in the MCAO + eNAMPT group (IL-1β: 54.15 ± 4.81 ng/mg vs. 34.48 ± 5.45 ng/mg, *P* < 0.001; TNF-α: 71.31 ± 3.34 ng/mg vs. 48.20 ± 6.35 ng/mg, *P* < 0.001). Treatment with SAL effectively suppressed this increase in both inflammatory factors (IL-1β: 17.83 ± 2.57 ng/mg; TNF-α: 19.47 ± 3.41 ng/mg; both compared to the MCAO + eNAMPT group, *P* < 0.001) (Figures 3A,B).

#### 3.2.3 SAL downregulates the expression of total NAMPT protein in the brain and restores NAD^+^ metabolic levels

**FIGURE 4:**
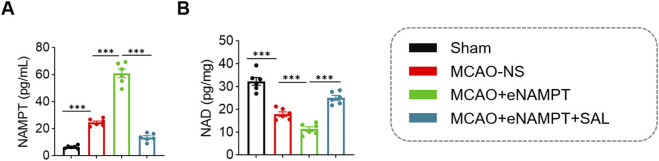
Comparison of NAMPT Content and NAD+ levels in brain tissue among the four rat groups. **(A)** Statistical analysis of NAMPT content in brain tissue across the four groups. **(B)** Statistical analysis of NAD+ content in brain tissue across the four groups. Data are presented as mean ± SD (n = 6 per group). ***P < 0.001.

We further examined the changes in total NAMPT protein and its metabolic product. Both cerebral ischemia and exogenous eNAMPT stimulation led to a significant upregulation of total NAMPT protein levels in brain tissue homogenates (MCAO-NS group: 24.55 ± 2.66 pg/mL, MCAO + eNAMPT group: 60.95 ± 7.79 pg/mL, both *P* < 0.001 compared to the Sham group (6.40 ± 1.11 pg/mL). Treatment with SAL significantly reduced these levels (13.51 ± 2.93 pg/mL, *P* < 0.001 vs. MCAO + eNAMPT group) (Figure 4A). At the metabolic level, cerebral ischemia and eNAMPT stimulation resulted in a significant decrease in NAD+ levels (MCAO + eNAMPT group: 11.32 ± 2.38 pg/mg vs. Sham group: 32.22 ± 4.26 pg/mg, *P* < 0.001). Notably, while reducing total NAMPT protein, SAL treatment also reversed the depletion of NAD+, significantly restoring its levels (24.98 ± 2.65 pg/mg, *P* < 0.001 vs. MCAO + eNAMPT group) (Figure 4B).

### 3.3 SAL inhibits eNAMPT-induced microglial injury and inflammatory response *in vitro*

To directly verify the effect of eNAMPT on microglia and the intervention efficacy of SAL at the cellular level, we employed the oxygen-glucose deprivation/reoxygenation (OGD/R) model in primary microglial cells.

#### 3.3.1 SAL attenuates eNAMPT-exacerbated OGD/R injury in cells

**FIGURE 5:**
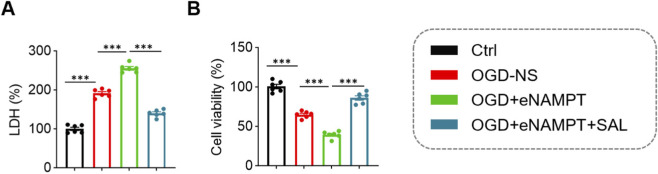
Comparison of cytotoxicity and viability among the four microglial cell groups. **(A)** Statistical analysis of microglial cytotoxicity. **(B)** Statistical analysis of microglial cell viability. Data are presented as mean ± SD (n = 6 per group). ***P < 0.001.

Compared to the normal control group (Ctrl), OGD/R treatment (OGD-NS group) significantly enhanced microglial cytotoxicity, as evidenced by a marked increase in lactate dehydrogenase (LDH) release (OGD-NS: 191.53% ± 10.38% vs. Ctrl: 100.05% ± 10.13%, *P* < 0.001), and concurrently significantly reduced cell viability (OGD-NS: 64.97% ± 4.29% vs. Ctrl: 100.65% ± 7.05%, *P* < 0.001). Exogenous eNAMPT stimulation further exacerbated OGD/R-induced cellular injury, leading to a more pronounced elevation in LDH release (255.42% ± 10.77% vs. OGD-NS: 191.53% ± 10.38%, *P* < 0.001) and a greater decline in cell viability (39.30% ± 4.39% vs. OGD-NS: 64.97% ± 4.29%, *P* < 0.001). Treatment with SAL significantly reversed the exacerbating effects of eNAMPT, reducing LDH release (139.90% ± 9.27% vs. OGD + eNAMPT group, *P* < 0.001) and restoring cell viability (86.12% ± 6.94% vs. OGD + eNAMPT group, *P* < 0.001) (Figures 5A,B).

#### 3.3.2 SAL inhibits eNAMPT-induced secretion of inflammatory cytokines in OGD/R-treated cells

**FIGURE 6:**
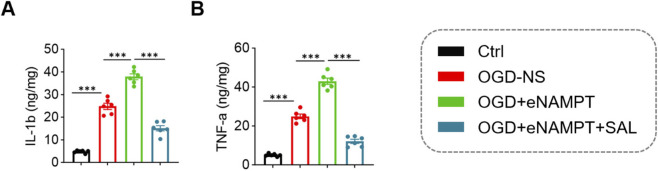
Comparison of inflammatory cytokine levels in microglial cell culture supernatants among the four groups. **(A)** Statistical analysis of IL-1β content in microglial cell culture supernatants across the four groups. **(B)** Statistical analysis of TNF-α content in microglial cell culture supernatants across the four groups. Data are presented as mean ± SD (n = 6 per group). ***P < 0.001.

Regarding the inflammatory response, compared to the Ctrl group, OGD/R treatment significantly induced the secretion of IL-1β and TNF-α from microglial cells (IL-1β: OGD-NS 24.79 ± 3.32 pg/mL vs. Ctrl 4.80 ± 0.60 pg/mL, *P* < 0.001; TNF-α: OGD-NS 24.98 ± 2.89 pg/mL vs. Ctrl 5.08 ± 0.69 pg/mL, *P* < 0.001). eNAMPT stimulation further significantly amplified this inflammatory response (IL-1β: OGD + eNAMPT group 37.86 ± 3.15 pg/mL vs. OGD-NS group 24.79 ± 3.32 pg/mL; TNF-α: 43.03 ± 3.95 pg/mL vs. 24.98 ± 2.89 pg/mL; both *P* < 0.001). Treatment with SAL effectively suppressed the excessive secretion of inflammatory cytokines driven by eNAMPT (IL-1β: 15.11 ± 2.88 pg/mL; TNF-α: 12.08 ± 2.60 pg/mL; both *P* < 0.001 compared to the OGD + eNAMPT group) (Figures 6A,B).

## 4 Discussion

This study reveals the neuroprotective effect of SAL in CIRI and preliminarily elucidates that its mechanism is closely associated with the regulation of the eNAMPT-mediated neuroinflammatory pathway. The results demonstrate that SAL not only significantly reduces cerebral infarction and neurological deficits but also effectively counteracts the injury exacerbated by exogenous eNAMPT. Its protective effects are accompanied by the inhibition of total NAMPT protein expression, restoration of NAD^+^ levels, and suppression of the neuroinflammatory response. These findings provide new experimental evidence for understanding the neuroprotective mechanisms of SAL and suggest that eNAMPT may serve as a key effector molecule in its pharmacological action.

### 4.1 NAMPT exhibits context-dependent dual functions in CIRI

As the rate-limiting enzyme in the NAD^+^ salvage synthesis pathway, iNAMPT has traditionally been regarded as crucial for maintaining cellular energy metabolism and redox homeostasis, playing a vital role in neuronal survival (Wang et al., 2019; Zhao et al., 2015). However, emerging evidence supports the concept of its context-dependent “dual function,” wherein eNAMPT acts as a DAMP with pro-inflammatory properties (Audrito et al., 2020; Martínez-Morcillo et al., 2021). Our results demonstrate that cerebral ischemia-reperfusion strongly induces the expression of total NAMPT in the brain, which coincides with a sharp increase in pro-inflammatory cytokines IL-1β and TNF-α (Figure 1). More importantly, through gain-of-function experiments, we confirmed both *in vivo* and *in vitro* that exogenous eNAMPT directly exacerbates post-ischemic cerebral infarction, neurological deficits, and microglial inflammatory responses (Figures 2–6). These findings position eNAMPT as a key pro-inflammatory factor and aggravating factor in cerebral ischemic injury, consistent with previous studies suggesting that eNAMPT can drive inflammation through non-enzymatic mechanisms (Zhao et al., 2013). As a DAMP, eNAMPT likely acts as an upstream “alarm” signal driving microglia toward a harmful pro-inflammatory phenotype (Fan et al., 2011; Martínez-Morcillo et al., 2021).

### 4.2 SAL counteracts eNAMPT-exacerbated brain injury and neuroinflammation

Based on the aforementioned pro-injury role of eNAMPT, we further evaluated the interventional effects of SAL. Treatment with SAL significantly reversed all injury phenotypes exacerbated by eNAMPT, including reduced cerebral infarction volume, improved neurological function (Figure 2), as well as rescued microglial viability and suppressed excessive secretion of inflammatory cytokines (Figures 5, 6). These observations demonstrate that SAL effectively antagonizes the pathogenic effects of eNAMPT, positioning it as a potential therapeutic agent targeting this inflammatory mediator.

### 4.3 The NAMPT/NAD^+^ paradox: selective modulation of the NAMPT system

A central finding of this study is the association between SAL treatment and reduced total NAMPT protein expression alongside restored NAD^+^ levels (Figures 4A,B). This observation appears counterintuitive given that NAMPT is the rate-limiting enzyme in the NAD^+^ salvage pathway (Rongvaux et al., 2002; Garten et al., 2015). However, several non-mutually exclusive hypotheses may reconcile this apparent paradox.

First, our measurements of total NAMPT in brain tissue homogenates do not distinguish between its two functionally distinct pools: intracellular iNAMPT, which is enzymatically active and essential for NAD^+^ synthesis, and extracellular eNAMPT, which acts primarily as a pro-inflammatory DAMP (Audrito et al., 2020; Martínez-Morcillo et al., 2021; Zhao et al., 2013). The reduction in total NAMPT protein observed after SAL treatment may predominantly reflect a decrease in the pathologically elevated eNAMPT pool, which is secreted by activated microglia and other cells during CIRI and contributes to neuroinflammation without directly participating in NAD^+^ synthesis (Li et al., 2008; Fan et al., 2011). Meanwhile, the essential NAD^+^-synthetic function of iNAMPT may be preserved or even enhanced.

Second, NAD^+^ levels are determined not only by its synthesis via NAMPT but also by its consumption through enzymes such as PARP-1, CD38, and sirtuins (Katsyuba et al., 2020; Imai and Guarente, 2016). Cerebral ischemia-reperfusion injury is known to trigger massive overactivation of PARP-1 in response to oxidative DNA damage, leading to rapid NAD^+^ depletion and subsequent energy failure (Endres et al., 1997; Liu et al., 2009). Therefore, the restoration of NAD^+^ levels by SAL could be attributable, at least in part, to inhibition of PARP-1 overactivation, thereby reducing NAD^+^ consumption. This mechanism would be consistent with SAL’s known antioxidant properties and its ability to attenuate oxidative stress (Fan et al., 2020; Han et al., 2015).

Third, SAL may enhance NAD^+^ levels through alternative biosynthetic pathways, such as the Preiss-Handler pathway using nicotinic acid or the *de novo* pathway from tryptophan (Katsyuba et al., 2020). Additionally, SAL’s documented ability to preserve mitochondrial function (Wang et al., 2019; Liu et al., 2018) could maintain cellular energy homeostasis and reduce the demand for NAD^+^-dependent repair processes, indirectly contributing to NAD^+^ preservation.

Notably, recent studies have demonstrated that SAL can activate PINK1/Parkin-mediated mitophagy to clear damaged mitochondria (Dong et al., 2026; Li and Chen, 2019), which may reduce mitochondrial ROS production and subsequent PARP-1 activation. Furthermore, Chai et al. (2024) reported that SAL interacts with key autophagy regulators including mTOR and SIRT1, suggesting a potential link between SAL’s effects on autophagy and NAD^+^ metabolism (Chai et al., 2024). Given that NAMPT protein degradation is closely associated with the autophagy-lysosome pathway (Dong et al., 2022), it is plausible that SAL promotes the selective autophagic degradation of pathologically elevated NAMPT (particularly eNAMPT), thereby reducing its pro-inflammatory effects while preserving or enhancing iNAMPT function through improved cellular energy status.

In summary, this nuanced perspective is critical: SAL may not be simply inhibiting NAMPT overall, but rather rebalancing the NAMPT system by curbing its harmful extracellular inflammatory functions while supporting its beneficial intracellular metabolic functions. This selective modulation of the NAMPT/NAD^+^ axis may represent a key upstream component of SAL’s multifaceted neuroprotective profile.

### 4.4 Integration with the known multi-target actions of SAL

These findings add to the known multi-target neuroprotective profile of SAL, which has been extensively documented to involve modulating microglial polarization, activating endogenous antioxidant and survival pathways, among others (Liu et al., 2018; Zhang et al., 2018; Fan et al., 2020). The association between SAL treatment and selective modulation of the NAMPT/NAD^+^ axis identified in this study may represent a novel upstream component of its complex pharmacological profile, specifically targeting the eNAMPT-driven inflammatory loop.

In recent years, SAL’s multi-target actions have been continually elucidated. For instance, it can promote BDNF signaling and neurogenesis by directly activating HSC70 (Lai et al., 2024), regulate glutamate metabolism in astrocytes (Zheng X. et al., 2024), promote endogenous neural regeneration via the Notch pathway and neurotrophic factors (Zheng J. et al., 2024), and protect organelles by activating PINK1/Parkin-mediated mitophagy (Dong et al., 2026). These studies enrich the mechanistic spectrum of SAL from perspectives such as promoting repair, regulating excitotoxicity, and clearing damaged mitochondria (Lai et al., 2024; Zheng X. et al., 2024; Zheng J. et al., 2024; Dong et al., 2026).

In contrast to these studies focusing on downstream protective pathways or cellular events, our research identifies an association between SAL treatment and changes in the metabolic-inflammatory hub molecule NAMPT. By establishing an “eNAMPT-aggravated injury” model, we demonstrated that SAL can counteract the pathological processes driven by this specific extracellular molecule, suggesting that modulation of aberrantly activated extracellular inflammatory signals contributes to SAL’s neuroprotective effects. Therefore, we propose that SAL functions through multiple interconnected mechanisms: the selective modulation of the NAMPT/NAD^+^ axis may represent an upstream event that curbs the initiation and amplification of injury, which then complements the downstream pro-neurogenic, anti-excitotoxic, and mitophagy-promoting effects discovered in other studies. Together, these various effects contribute to the comprehensive neuroprotective profile of SAL.

### 4.5 Considerations regarding route of administration and clinical translation

While the ICV administration employed in this study was essential for establishing a direct mechanistic link between SAL and central NAMPT/neuroinflammation by ensuring precise drug delivery and eliminating peripheral interference, it inherently differs from clinically relevant systemic routes and limits direct translational extrapolation. However, the robustness of our central mechanism is supported by a substantial body of evidence demonstrating that SAL can effectively cross the BBB. Numerous *in vivo* studies utilizing systemic administrations, such as intraperitoneal injection or oral gavage, have consistently reported significant neuroprotective effects of SAL in cerebral ischemia models (Fan et al., 2020; Zhang et al., 2021; Liu et al., 2018). Therefore, our ICV-based findings provide critical mechanistic insights that help explain the therapeutic benefits observed with systemic SAL administration. They suggest that the neuroprotection conferred by peripherally administered SAL is, at least in part, attributable to its direct action on the central NAMPT/NAD^+^ axis and microglial inflammation. Future investigations should integrate systemic administration models, such as intravenous or intraperitoneal injection, to further validate the clinical potential of targeting the NAMPT pathway with SAL and to comprehensively assess its pharmacokinetics and dose-response relationships in a therapeutically relevant context.

### 4.6 Limitations and future directions

This study has several limitations that should be acknowledged. First, the data demonstrate a correlational reduction in total NAMPT protein following SAL treatment, but no binding assays, transcriptional analysis, or upstream signaling data have been provided to establish direct molecular targeting. Crucially, our experimental approach does not directly distinguish between the iNAMPT and eNAMPT pools in the tissue homogenate measurements. The reduction in total NAMPT could be due to decreased eNAMPT secretion, reduced expression of iNAMPT in specific cell types, or enhanced degradation of both forms. The specific molecular mechanisms through which SAL influences the balance between iNAMPT and eNAMPT—whether at the level of transcription, translation, protein secretion, or degradation—have not been elucidated.

Second, regarding the NAMPT/NAD^+^ paradox, we acknowledge that our experimental approach does not provide compartment-specific resolution of NAMPT pools or direct measurements of NAD^+^ consumption rates. Future studies should therefore: (1) develop methods to specifically quantify iNAMPT and eNAMPT separately; (2) directly assess PARP-1 activity and NAD^+^ consumption; (3) evaluate the contribution of alternative NAD^+^ synthesis pathways; and (4) investigate whether SAL promotes selective autophagic degradation of eNAMPT.

Third, the direct effects on downstream key effector molecules (such as SIRT1) remain to be validated. Fourth, our *in vitro* investigation of microglia primarily focused on the release of pro-inflammatory cytokines (TNF-α and IL-1β) as functional endpoints. While this approach successfully demonstrated that eNAMPT directly activates microglial inflammation and that SAL counteracts this effect, it did not include a comprehensive phenotypic characterization of microglial polarization states (e.g., M1 vs. M2 markers such as iNOS, CD206). This limits our ability to fully interpret the functional transition of microglia and whether SAL’s protective effects involve a shift from a pro-inflammatory (M1) to an anti-inflammatory/repair (M2) phenotype.

Based on our findings, future research should further explore: ① Utilizing conditional gene knockout technology (particularly in microglia) to verify whether the effects of SAL depend on changes in cell-specific NAMPT expression and its subsequent release as eNAMPT. ② Investigating the molecular mechanisms by which SAL influences the balance between iNAMPT and eNAMPT, including transcriptional regulation, post-translational modifications, and protein stability and secretion. ③ Systematically evaluating the overall impact of SAL on the NAD^+^ metabolic network (including synthesis, consumption, and sensing) to comprehensively elucidate the mechanisms by which it may restore metabolic homeostasis while selectively dampening eNAMPT-mediated inflammation. ④ Incorporating detailed microglial polarization assays to determine whether SAL modulates the M1/M2 balance through the NAMPT pathway. Investigating whether causal or synergistic relationships exist between SAL’s effects on the NAMPT system and its known effects, such as activating HSC70 (Lai et al., 2024) and modulating mitophagy (Dong et al., 2026), will be key to understanding how its multi-target network is integrated.

## 5 Conclusion

In conclusion, this study demonstrates that the neuroprotective effects of SAL against cerebral ischemia-reperfusion injury are associated with downregulation of pathological total NAMPT expression (likely reflecting a reduction in pro-inflammatory eNAMPT), restoration of NAD^+^ homeostasis (indicating preserved iNAMPT function), and inhibition of microglia-mediated neuroinflammation. These findings provide experimental evidence linking SAL treatment to a selective modulation of the NAMPT/NAD^+^ axis, suppressing its detrimental extracellular inflammatory role while preserving its beneficial intracellular metabolic function. This study suggests that targeting eNAMPT-mediated neuroinflammation may be a potential effector mechanism in the pharmacological actions of SAL, offering a novel pharmacological basis for understanding its clinical application and providing important theoretical insights for developing new strategies to treat ischemic stroke by targeting cerebral metabolic-inflammatory homeostasis.

## Data Availability

The raw data supporting the conclusions of this article will be made available by the authors, without undue reservation.
